# Multichannel Discriminative Detection of Explosive Vapors with an Array of Nanofibrous Membranes Loaded with Quantum Dots

**DOI:** 10.3390/s17112676

**Published:** 2017-11-20

**Authors:** Zhaofeng Wu, Haiming Duan, Zhijun Li, Jixi Guo, Furu Zhong, Yali Cao, Dianzeng Jia

**Affiliations:** 1 School of Physics Science and Technology, Xinjiang University, Urumqi 830046, China; wzf911@mail.ustc.edu.cn (Z.W.); dhm@xju.edu.cn (H.D.); 2 Key Laboratory of Energy Materials Chemistry, Ministry of Education, Key Laboratory of Advanced Functional Materials, Xinjiang University, Urumqi 830046, China; jxguo1012@163.com (J.G.); zhfuru@shzu.edu.cn (F.Z.)

**Keywords:** discriminative detection, explosive vapors, sensor array, nanofibrous membranes, quantum dots

## Abstract

The multichannel fluorescent sensor array based on nanofibrous membranes loaded with ZnS quantum dots (QDs) was created and demonstrated for the discriminative detection of explosives. The synergistic effect of the high surface-to-volume ratio of QDs, the good permeability of nanofibrous membranes and the differential response introduced by surface ligands was played by constructing the sensing array using nanofibrous membranes loaded with ZnS QDs featuring several surface ligands. Interestingly, although the fluorescence quenching of the nanofibrous membranes is not linearly related to the exposure time, the fingerprint of each explosive at different times is very similar in shape, and the fingerprints of the three explosives show different shapes. Three saturated vapors of nitroaromatic explosives could be reliably detected and discriminated by the array at room temperature. This work is the first step toward devising a monitoring system for explosives in the field of public security and defense. It could, for example, be coupled with the technology of image recognition and large data analysis for a rapid diagnostic test of explosives. This work further highlights the power of differential, multichannel arrays for the rapid and discriminative detection of a wide range of chemicals.

## 1. Introduction

One pressing concern in antiterrorism and homeland security is explosive detection [1,2,3]. Among the current explosive detection methods, fluorescent sensing represents one of the most promising approaches for trace explosives detection due to possible short response time, excellent sensitivity, simplicity and low cost [1,4,5]. Great efforts regarding fluorescent materials have been made in order to conveniently, quickly and effectively detect explosives. Conjugated polymers [1,6,7], organic dyes such as porphyrinoid and dendrimer [3,8], and microporous metal-organic frameworks [9,10,11] are proven to be high-performance fluorescent sensing materials, but their application is always limited by costly and cumbersome syntheses [4,12]. In comparison with organic dyes such as rhodamine, the fluorescent quantum dots (QDs) are 20 times as bright and 100 times as stable against photobleaching, showing better potential applications in various fields [13]. Recently, considerable progress has been made in the field of explosive detection based on the fluorescent sensors of QDs [14,15,16] with a high surface area-to-volume ratio. For example, Itamar Willner reported on the use of chemically modified CdSe/ZnS QDs as fluorescent probes for the detection of trinitrotoluene (TNT) or trinitrotriazine (RDX). The sensitivities of the QDs sensors are controlled by the electron donating properties of the capping layer that modifies the particles, thus allowing the quantitative analysis of the explosive substrates. Bingxin Liu [17] constructed the dual-luminescence-emission probe of CdTe/ZnS QDs and a novel ligand containing 8-hydroxyquinoline for selective sensing of the picric acid (PA) in aqueous solution. The study provides a new insight into highly selective fluorescent sensing of the nitroaromatic explosive PA with a detection limit of 9 nm. Leyu Wang [18] prepared ZnS:Mn^2+^@allyl mercaptan nanocomposites through novel light-induced in situ polymerization to detect sensitively and selectively nitroaromatic explosives. The fluorescent probe can linearly detect TNT and PA in the range of 0.01–0.5 μg/mL and 0.05–8.0 μg/mL, respectively, barely interfered with by other nitroaromatics such as 2,4-dinitrotoluene (DNT) and nitrobenzene (NB). Neelotpal Sen Sarma [19] synthesized Poly(vinyl alcohol)-grafted polyaniline (PPA) and its nanocomposites with 2-mercaptosuccinic acid (MSA)-capped CdTe QDs and with MSA-capped CdTe/ZnS QDs via a single step free radical polymerization reaction. The detection limits of PPA, MSA-capped CdTe, and MSA-capped CdTe/ZnS QDs for PA in aqueous solution are found to be 23, 1.6, and 0.65 nm, respectively, which are remarkably low. William J. Peveler [7] reported a multichannel array based on multicolored, fluorescent CdTe/ZnS QDs with surface functionalities for the detection of explosives in a rapid single fluorometric test. Pattern analysis of the fluorescence quenching data allows for explosive detection and identification, and five explosives, DNT, TNT, tetryl, RDX and pentaerythritol tetranitrate (PETN), are detected and differentiated in a multichannel fluorescent platform. Zhongping Zhang [20] embedded the red-emitting CdTe QDs in silica nanoparticles and covalently linked the green-emitting CdTe QDs to the silica surface, respectively, to form a dual-emissive fluorescent hybrid nanoparticle. The fluorescence of red QDs in the silica nanoparticles stays constant, whereas the green QDs functionalized with polyamine can selectively bind TNT, leading to the green fluorescence quenching due to resonance energy transfer. The variations of the two fluorescence intensity ratios display continuous color changes from yellow-green to red upon exposure to different amounts of TNT. 

As can be seen from the above representative works, a series of fluorescent probes containing QDs have also been applied for the detection of explosives, but mainly limited to explosive solutions and/or particulates (through direct contact) [2,3,21]. Moreover, the vast majority of studies have not tried to discriminate between multiple types of explosive. Compared to the detection in solution and solid phases, the detection of explosives in vapor phase is more challenging and desired since most of them have substantially low volatility [5,22]. Furthermore, QDs in solutions are easy to agglomerate and precipitate, and the long-term stability is poor, which is not conducive to the effective detection of explosives. Electrospinning has become a simple, cost-effective and versatile technique for the preparation of nanomaterial films with high porosity and flexibility, which has great potential for enhanced explosive detection [23,24,25]. The combination of QDs and electrospun fibers may provide a novel possibility for the low-cost, sensitive, discriminative detection of explosives in vapor phase. Lastly, the preparation of some QDs, such as CdTe and InP, usually needs oil bath with high reaction temperature and they are easily oxidized in air, while ZnS QDs can be prepared in aqueous solution at room temperature and have good oxidation resistance, which can be easily combined with electrospun polymer fibers for the detection of gaseous explosives.

Herein we report a fluorescent sensor array based on nanofibrous membranes loaded with ZnS QDs followed by the modification of several surface ligands for the detection of explosive vapors. The synergistic effect of the high surface-to-volume ratio of QDs, the good permeability of nanofibrous membranes and the differential response owing to the surface ligands was played in the sensing system. The sensing system is designed to respond to a range of explosives through supramolecular interactions, such as host-guest binding and electrostatics, causing fluorescence quenching of the QDs, to create an analytical fingerprint for the sensitive, quick, discriminative detection of explosive vapors at room temperature.

## 2. Materials and Methods 

### 2.1. Materials

Analytically pure sodium sulfide, zinc acetate, manganese acetate, lysine, L-cysteine, trifluoroacetyl lysine, L-cysteine hydrochloride were purchased from Aladdin Reagent Co., Ltd., (Los Angeles, CA, USA). Nitrobenzene (NB) was purchased from Sinopharm Chemical Reagent Co., Ltd., (Shanghai China). Picric acid and 2, 4-dinitrotoluene were used as received from national standard substance Center. Dimethylformamide (DMF) and methanol solution of 2, 4, 6-Trinitrotoluene (1000 μg/mL, TNT) was purchased from Sinopharm Chemical Reagent Co., Ltd., (Shanghai China), and TNT was recrystallized to produce saturated vapor at room temperature. Polyurethane (PU, *M*w = 200,000) was supplied by Anhui Amway synthetic leather Co., Ltd., (Hefei China).

Caution: The highly explosive TNT and PA should be used with extreme caution and handled only in small quantities.

### 2.2. Synthesis of Mn^2+^-Doped ZnS QDs

Sodium sulfide was used at the mole amount equal to that of zinc acetate. Typically, 50 mmol of zinc acetate was dissolved in mixed solution of ethanol and deionized water (volume ratio = 1:1). An amount of 2.5 mmol of manganese acetate was added into the above solution, and the mixture was ultrasonicated for 20 min at room temperature. After the mixture solution was refluxed in a flask under nitrogen, 10 mL of aqueous solution containing 50 mmol of sodium sulfide was added dropwise into the reaction system, and the mixture was vigorously stirred for 5 h. The resultant Mn^2+^ doped ZnS QDs were centrifuged and washed with deionized water several times.

### 2.3. Preparation of Electrospun Nanofibrous Membrane 

The preparation of PU electrospun nanofibrous membrane was performed as follows: an electrospun solution was initially prepared by dissolving 1.2 g of PU and 0.18 g of zinc acetate in 10 mL of DMF. The prepared solution was transferred into a 10 mL syringe mounted on the electrospinning apparatus (SS-2535H). A high-voltage power supply generated direct-current voltage up to 18 kV. The electrospun solution was fed at a constant rate of 0.8 mL/h by a syringe pump. Nanofibers were collected on aluminum foil with a collection time of 120 min, and were then dried in an oven at 50 °C for 12 h to remove the residual organic solvent. The electrospun membranes were recorded as PU-0.

### 2.4. Preparation of Fluorescent Nanofibrous Membranes

The preparation of membranes loaded with ZnS QDs was prepared as follows. 

First, 50 mmol of zinc acetate was dissolved in 50 mL mixed solution of ethanol and deionized water (volume ratio = 1:1). Second, 2.5 mmol of manganese acetate was added into the above mixed solution, followed by a ultrasonic dispersion process for 20 min and a reflux for 1 h under nitrogen protection. Third, the PU-0 membranes were immersed in the mixed solution with slow stirring and the reaction device was kept in an ice water mixture for 5 h. Fourth, 10 mL of aqueous solution containing 50 mmol of sodium sulfide was added dropwise into the reaction system, and stirred for 4, 8, and 12 h, respectively. Last, the PU-0 membranes loaded with ZnS QDs were washed with deionized water several times and the resultant electrospun membranes corresponding to 4, 8, and 12 h were recorded as PU-1, PU-2 and PU-3, respectively. 

The preparation of membranes including ZnS QDs was performed as follows. 

First, an electrospun solution was initially prepared by dissolving 0.25 g of ZnS QDs as fluorescence probes and 1.2 g of PU as the supporting polymer in 10 mL of DMF. Second, the prepared solution was transferred into a 10 mL syringe mounted on the electrospinning apparatus (SS-2535H). Third, the electrospun solution was fed at a constant rate of 0.8 mL/h by a syringe pump with a 20 kV direct-current voltage. At last, nanofibers were collected on aluminum foil with a collection time of 120 min, and then dried in an oven at 50 °C for 12 h to remove the residual organic solvent. The sample was recorded as PU-4.

### 2.5. Surface Modification of Fluorescent Nanofibrous Membranes

For the further surface modification, an amount of 0.25 mmol of lysine, cysteine, trifluoroacetyl lysine, cysteine hydrochloride (Structural formula shown in Figure S1) was dissolved in 50 mL of mixed solution of ethanol and deionized water (volume ratio = 1:1), respectively. Then the fluorescent membranes (PU-1) were immersed in the above mixture solutions with slow stirring for 24 h, respectively. Mercapto groups of cysteine and cysteine hydrochloride tightly attached onto the surface of the QDs due to the excess of metal ions with respect to sulfide ions at the surface of the QDs [26]. As shown in Figure S2, the infrared bands located at about 3500 cm^–1^ are due to the –OH stretching on the surfaces of ZnS QDs. The strong bands located at about 3500 cm^-1^ indicates the presence of a large number of hydroxyl on the surface of ZnS QDs. Similarly, lysine and trifluoroacetyl lysine also tightly attached onto the surface of the QDs due to the interaction between carboxyl groups of lysine and trifluoroacetyl lysine and hydroxyl groups at the surface of the QDs [27]. The modified fluorescent membranes were washed with ethanol several times to remove the residue and were dried at 50 °C for use. PU-1 membranes treated by lysine, cysteine, trifluoroacetyl lysine and cysteine hydrochloride were recorded as PU-1L, PU-1C, PU-1TL and PU-1CH, respectively. 

### 2.6. Quenching Tests of Fluorescent Nanofibrous Membranes towards Nitroaromatic Explosives 

Before quenching tests, the fluorescent nanofibrous membranes were placed under an ultraviolet (UV) lamp for 12 h to make the fluorescence intensity stable. Small granules of nitro analytes were placed on the bottom of a sealed testing box with four quartz windows and a slot. Meanwhile, a small amount of cotton gauze was tucked into the testing box to help maintain a constant vapor pressure of analyte. After the slot was sealed, the vapor of nitro compound was up to saturation in the testing box for 48 h at room temperature (25 °C). The nanofibrous membranes were fixed onto the sample shelf tightly matched with the slot of the testing box and promptly inserted into the testing box filled with saturated vapor through the slot. The evolution of fluorescence spectra was recorded for specific time intervals after exposing the films to the vapor of analytes.

### 2.7. Characterization 

Steady-state luminescence spectra were acquired under excitation at 300 nm on a Hitachi F4600 luminescence spectrometer (Hitachi, Tokyo Japan). The UV-Vis absorbance spectra were recorded with a UV-3900H spectrometer (Hitachi, Japan). Thermal gravimetric analysis (TGA) was carried out using a 200F3 thermal gravimetric analyzer (Netzsch, Gebrüder Germany) at a heating rate of 10 °C/min under air condition. Transmission electron microscope (JEM-2100F) and field emission scanning electron microscopy (FE-SEM, S-4800, Hitachi, Japan) was used to characterize morphology of samples. Contact angles of the films with deionized water drop were measured with a contact angle goniometer (G-1, Erma) at room temperature. Infrared spectra of the ZnS QDs was recorded on a VERTEX 70 Fourier transform infrared spectrometer (Bruker, Karlsruhe Germany).

## 3. Results and Discussion

### 3.1. Preparation and Surface Modification of Fluorescent Nanofibrous Membranes

**Figure 1:**
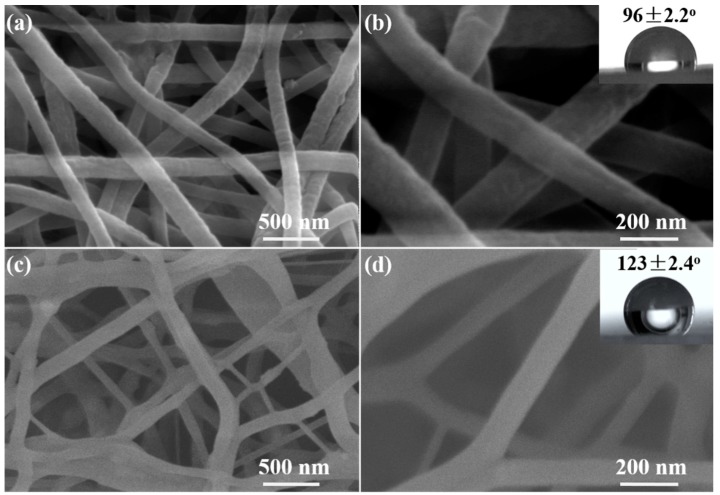
Scanning electron microscopy (SEM) images with different magnification of (**a**,**b**) PU-0, (**c**,**d**) polyurethane (PU)-4 nanofibrous membrane, the insets in (**b**,**d**) show images of water drops and the corresponding contact angles.

As shown in Figure 1a,b, the PU-0 nanofibrous membrane is consisted of fibers between 150 and 200 nm in diameter, and their surfaces are basically smooth. Similarly, the PU-4 nanofibrous membrane is consisted of smooth fibers between 50 and 250 nm in diameter, and there are no ZnS QDs on the fiber surfaces (Figure 1c,d), indicating that the ZnS QDs are mainly distributed in the inner part of the nanofibers. To further analyze the surface properties of two kinds of fiber membranes, the static contact angle test was performed. Their static contact angles of PU-0 and PU-4 are 96 ± 2.2° and 123 ± 2.4°, respectively (inserts in Figure 1). While the static contact angle of pure PU nanofibrous membrane is about 125° (Figure S3), which is very close to that of PU-4. The result indicates that the incorporation of ZnS QDs has not changed significantly the surface properties of PU-4 compared to the pure PU nanofibrous membrane. The surface properties of PU-0 are changed significantly because of the incorporation of hydrophilic zinc acetate, which is conducive to the growth or load of the hydrophilic QDs on the PU-0 membrane.

**Figure 2:**
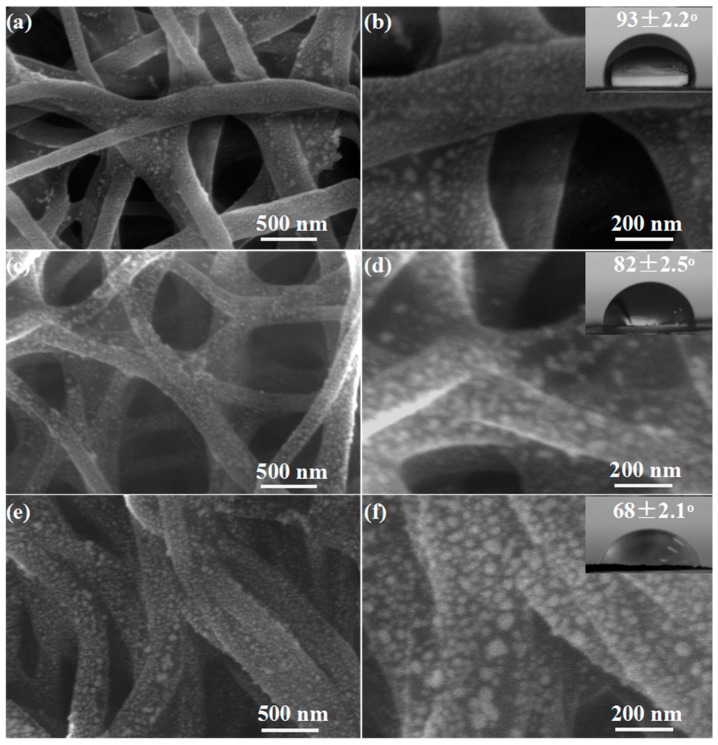
SEM images with different magnification of (**a**,**b**) PU-1, (**c**,**d**) PU-2, and (**e**,**f**) PU-3 nanofibrous membrane, the insets in (**b**,**d**,**f**) show images of water drops and the corresponding contact angles.

As shown in Figure 2a–f, compared with the PU-0, the fiber diameter of PU-1, PU-2 and PU-3 membranes loaded with ZnS QDs did not change significantly because of the tiny particle size, but the surfaces of fibers become rough. Further observations show that the surfaces of the fibers are loaded with a layer of ZnS QDs and more and more ZnS QDs are loaded on the fiber surface from PU-1 to PU-3. The static contact angles of PU-1, PU-2 and PU-3 also have obviously changed, reaching 93, 82 and 68°, respectively. The significant change of surface properties of nanofibers should be attributed to the hydrophilicity of the hydroxyl groups of ZnS surfaces (Figure S2). These results indicate that ZnS QDs are effectively loaded on the fiber surfaces, which could be illustrated by a schematic diagram. As a result, the rough topography of fibers loaded with ZnS QDs shown in Figure 2 is formed. As shown in Figure S4, when PU-0 membranes are immersed in the mixture of manganese and zinc ions with slow stirring, the zinc ions in the solution are adsorbed on the surface of the nanofibers owing to the high surface-to-volume ratio of nanofibers, forming a large number of reactive sites. With the increase of soaking time, the zinc ions in the nanofiber inner migrate to the surface of the fiber and also form the reactive sites. At the same time, the ice bath is used to control the reaction temperature, thus controlling the reaction rate. When the aqueous solution of sodium sulfide is slowly added, the sulfide ion combines with the reactive sites of the fiber surface to form ZnS QDs and ZnS QDs continue to grow or agglomerate together with the increase of reaction time, forming larger QDs agglomerates on the fiber surfaces.

**Figure 3:**
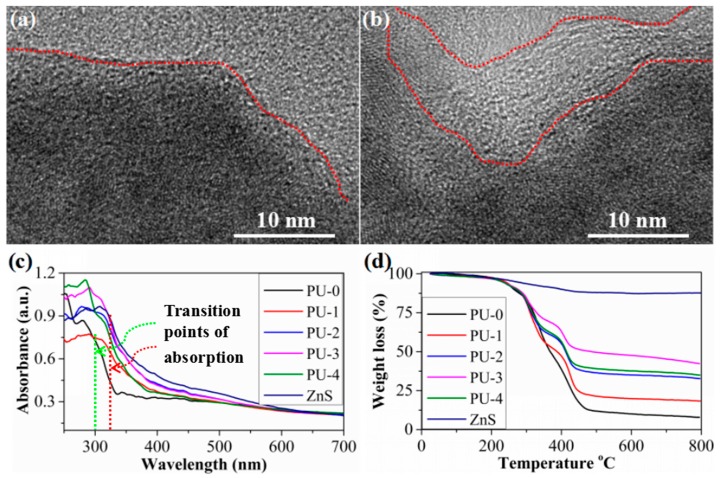
Transmission electron microscope (TEM) images of ZnS quantum dots (QDs) (**a**) on the fiber surfaces of PU-2, (**b**) in the fiber inner of PU-4 (the red dotted curves in Figure 4a,b show the boundary between ZnS and the polymer layer), (**c**) ultraviolet (UV)-vis absorption spectrum (the green and red dotted lines show the transition points of UV-Vis absorption of the PU-0 and ZnS), (**d**) thermal gravimetric analysis (TGA) curves of samples.

In order to qualitatively and quantitatively determine the amount of ZnS QDs on the nanofiber surfaces, TEM, UV-Vis absorption and TGA were performed. As shown in Figure 3a, ZnS QDs are in a state of agglomeration and there is no polymer layer on the surface of ZnS QDs for PU-2. For PU-4, although ZnS QDs are also in a state of agglomeration because of the large amount of ZnS addition, ZnS QDs are coated with a polymer layer of about several nanometers in thickness (as shown by the red dotted curve in Figure 3b). The results also show that ZnS QDs are distributed in the inner part of the PU-4 nanofibers, which is consistent with the observation of SEM in Figure 1. As shown by the red and green dotted lines in Figure 3c, the transition points of UV-Vis absorption of PU-0 and ZnS QDs are about 300 and 325 nm, respectively. Also, the absorption intensity of ZnS QDs is obviously higher than that of the PU-0 in the range of 240-500 nm. It is worth pointing out that the transition points of UV-Vis absorption of PU-1, PU-2, PU-3 and PU-4 are very close to that of ZnS, showing the similar absorption characteristics to ZnS. The increase of absorption intensity in the ultraviolet region from PU-1 to PU-3 indicate that the amount of ZnS loaded on the nanofiber surface also increase with the increase of reaction time. This result is further demonstrated by TGA tests. TGA was performed in a nitrogen atmosphere to prevent the oxidation of ZnS at elevated temperatures. As shown in Figure 3d, the residue of all the samples is basically stable after 500 °C, and the residue weight of PU-0, PU-1, PU-2, PU-3, PU-4 and ZnS is 7.8, 18.5, 33.3, 42.5, 35.2 and 87.6% at 800 °C, respectively. Compared with PU-0, the residue weight of PU-1, PU-2, PU-3 and PU-4 is increased by about 10.7, 25.5, 34.7 and 27.4%, respectively, due to the introduction of ZnS QDs. The TGA results also illustrate the effective load of ZnS on the nanofiber surfaces, facilitating the preparation of fluorescent nanofibrous membrane array for explosive sensing.

### 3.2. Fluorescent Nanofibrous Membrane Array for Explosive Sensing

**Figure 4:**
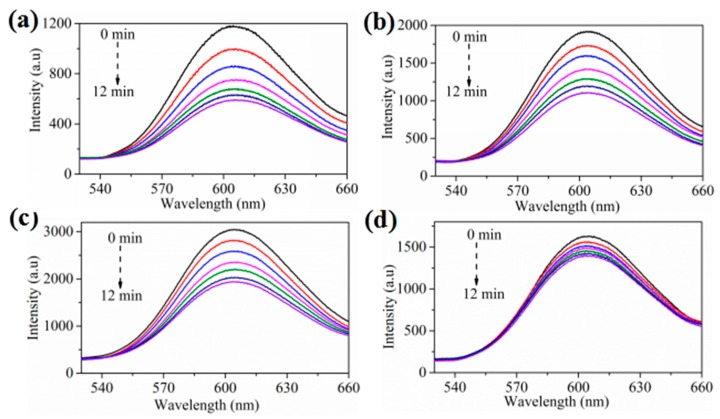
Time-dependent fluorescence emission spectra of the nanofibrous membranes (**a**) PU-1, (**b**) PU-2, (**c**) PU-3, and (**d**) PU-4 upon exposure to 4 ppb of trinitrotoluene (TNT) vapor.

In order to evaluate the quenching effect of fluorescent nanofibrous membranes with different loading amounts of ZnS towards nitroaromatic explosive vapors, time-dependent fluorescence emission spectra were performed. As shown in Figure 4, the fluorescence intensity of PU-1, PU-2 and PU-3 increase significantly with the increase of loaded ZnS before exposure to TNT vapor, reaching about 1180, 1900 and 3000. Because the loading amounts of ZnS in PU-4 is close to that of PU-2, the fluorescence intensity of PU-4 is close to that of PU-2, reaching about 1700 due to the coating of polymer layers. And one can see that from Figure 4 and Figure S5, the fluorescence quenching rate gradually decreases from PU-1 to PU-3 due to the increase of loaded ZnS. It is worth pointing out that although PU-4 does not have the most loads of ZnS, the fluorescence quenching rate of PU-4 is the slowest, which should be attributed to the coating of ZnS by the polymer layer. The coating of polymer layer effectively prevents the contact of ZnS and TNT vapor, causing the slow quenching rate. Based on the quenching performances of the above fluorescent membranes, PU-1 is selected as an ideal fluorescent sensing membrane for the detection of nitroaromatic explosive vapors. Furthermore, the reproducibility of fluorescent nanofibrous membranes were evaluated. The fluorescence quenching efficiencies (presented as (1–I/I_o_), where I_o_ is the initial fluorescence intensity in the absence of analyte, I is the fluorescence intensity in the presence of analytes) of the above four kinds of membranes towards TNT vapor as a function of time among three batches were comparable (Figure S5). The results indicated that the fluorescent nanofibrous membranes show satisfactory reproducibility for application to the detection of explosives. 

**Figure 5:**
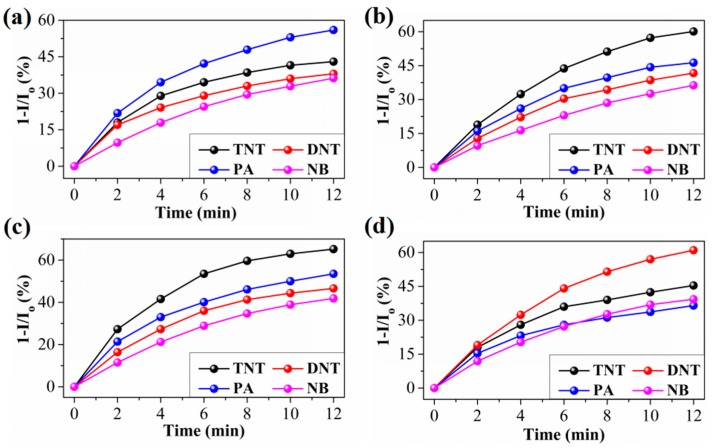
Variation of the quenching percentage for (**a**) PU-1C, (**b**) PU-1CH, (**c**) PU-1L and (**d**) PU-1TL as a function of the exposure time to the saturated air of TNT, 2,4-dinitrotoluene (DNT), picric acid (PA) and nitrobenzene (NB) at room temperature.

In order to realize the differential quenching of fluorescent membranes towards explosives, PU-1 membranes are modified by lysine, cysteine, trifluoroacetyl lysine and cysteine hydrochloride, recorded as PU-1L, PU-1C, PU-1TL and PU-1CH, respectively. After modification, mercapto groups of cysteine and cysteine hydrochloride tightly attached onto the surface of the QDs due to the excess of metal ions with respect to sulfide ions at the surface of the QDs [26]. Similarly, lysine and trifluoroacetyl lysine also tightly attached onto the surface of the QDs due to the interaction between carboxyl groups of lysine and trifluoroacetyl lysine and hydroxyl groups at the surface of the QDs [27]. When fluorescent membranes are exposed to the explosive vapors, the ligand should bind the explosive, and an electron-transfer mechanism between the QD and the electron-deficient explosive causes QD fluorescence quenching. Figure 5 shows the variation of the quenching percentage as a function of the exposure time to TNT, DNT, PA and NB. The response curves of fluorescent membranes towards different analytes are displayed in Figure S6 and S7. As for PU-1C, after about 12 min, the quenching percentage is ~43, 38, 56 and 36% (Figure 5) for saturated TNT (4 ppb) [26], DNT (180 ppb [28] or 200 ppb [26]), PA (0.0077 ppb) [26,28] and NB (3 × 10^5^ ppb [28] or 4 × 10^5^ ppb [26]) vapors at 25 °C, respectively. Because the vapor pressures of TNT and DNT are about 520 and 2.3~2.6 × 10^4^-fold that of PA, respectively, the quenching percentage for PA is thus surprisingly larger than that expected from the relative vapor pressure of these analytes. In terms of molecular structure (Figure S8), PA is a stronger acid than TNT, and a stronger acid-base pairing interaction thus occurs between PA and amino ligands, resulting in the formation of PA anions at the surface of amine-capped ZnS QDs [26]. However, it is well known that the DNT molecules with two nitro groups are much weaker Lewis acids and electron acceptors than TNT molecules. This suggests that it is less likely to form a Mesienheimer complex with the amine for DNT by the relatively weak basic amine groups. Therefore, the enhanced sensitivity toward PA vapor originates from the extremely strong adsorption of PA species at the amino of QDs and the larger quenching efficiency due to the high electron-accepting ability. Moreover, the high surface-to-volume ratio of QDs [29,30] and the good permeability of nanofiber membranes [5,31,32] are further advantageous to the enhancement of the interaction between nitroaromatic explosive vapors and the amino ligands, helping maximize the quenching efficiency. The fluorescent membranes of PU-1CH show the differential responses towards explosives owing to the different molecular structures of ligands, after 12 min the quenching percentage was ~60, 42, 47 and 36% for saturated TNT, DNT, PA and NB vapors at 25 °C, respectively. Lysine has two amino groups, but the response of PU-1L is not as we expected it to be. The quenching percentage towards TNT, DNT and PA vapors does not increase simultaneously, only reaching 36 and 45% for DNT and PA, respectively. Trifluoroacetyl lysine has an amino group, an amino group and three substituted fluorine atoms and the quenching percentage of PU-1TL towards saturated TNT, DNT, PA and BN vapors at 25 °C is ~45, 61, 37 and 39%, respectively. It is possible that the high quenching percentage towards DNT should be attributed to the polarity interaction of DNT and trifluoroacetyl lysine brought by the fluorine atoms. It is worth noting that although the vapor pressure of NB is much higher than TNT and PA, the fluorescence quenching efficiency is still lower than TNT and PA. This should be attributed to the weaker electron-withdrawing ability of BN with only one electron-withdrawing nitro group, compared with TNT and PA with three electron-withdrawing nitro groups. As a result, the differential quenching of fluorescent membranes towards nitroaromatic explosives is basically achieved by the surface modification of QDs, which lays a good foundation for the recognizable detection of nitroaromatic explosives.

### 3.3. Discriminative Detection Based on Fluorescent Nanofibrous Membrane Array

**Figure 6:**
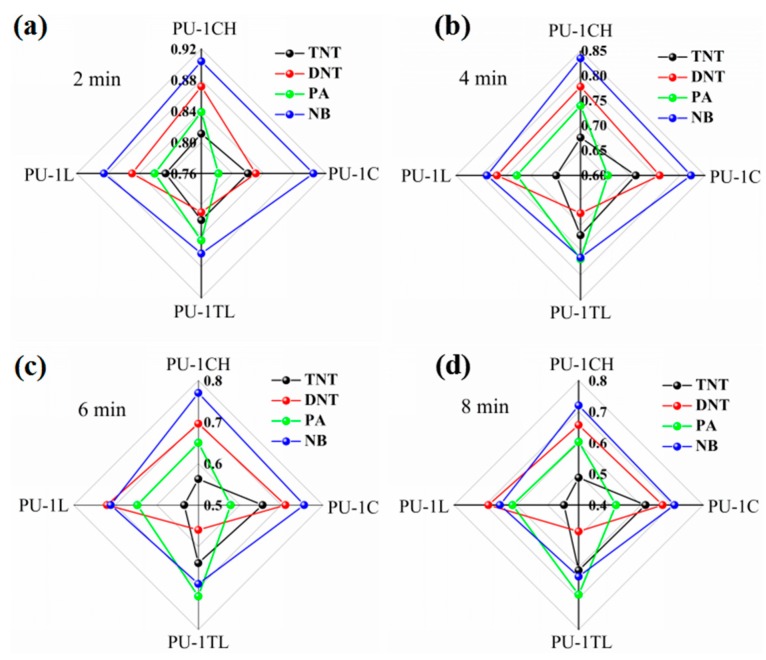
Fingerprints of three nitroaromatic explosives according to the quenching percentage of the sensing array based on fluorescent membranes as a function of time (**a**) 2 min, (**b**) 4 min, (**c**) 6 min, (**d**) 8 min.

In order to evaluate the recognizable detection of the fluorescent sensor array towards explosives, the quenching responses at different times of the sensor array including four fluorescent sensors were used to obtain the fingerprints of four nitroaromatic explosives. Figure 6 shows the transformation of fingerprints of four nitroaromatic explosives along with time. Interestingly, although the quenching of the fluorescent membranes is not linearly related to the exposure time (Figure 6), the fingerprint of each explosive at different times is very similar in shape and the fingerprints of the four explosives show different shapes. For example, the fingerprints of TNT, DNT, PA and NB corresponding to 2 min are not significantly different from those corresponding to 6 or 8 min, so it is possible to discriminate the explosives based on the fingerprints at 2 min or even shorter time. Thus, the fingerprints of the four explosives are clearly different because of the differential quenching of fluorescent membranes towards nitroaromatic explosives, which means that the recognizable detection of explosives by the fluorescent sensor array has been realized. More importantly, because of the stable relationship between fingerprints and time, the detection time of explosives can be effectively shortened, so as to achieve the recognizable detection of explosives in a shorter period of time. Or it can be used to determine unidentified explosives by monitoring the changes in the fingerprints along with time. It could, for example, be coupled with the technology of image recognition and large data analysis for a rapid diagnostic test of explosives, which is very important for the rapid and recognizable detection of explosives.

### 3.4. Homogeneity, Stability and Recoverability of Fluorescent Nanofibrous Membranes

**Figure 7:**
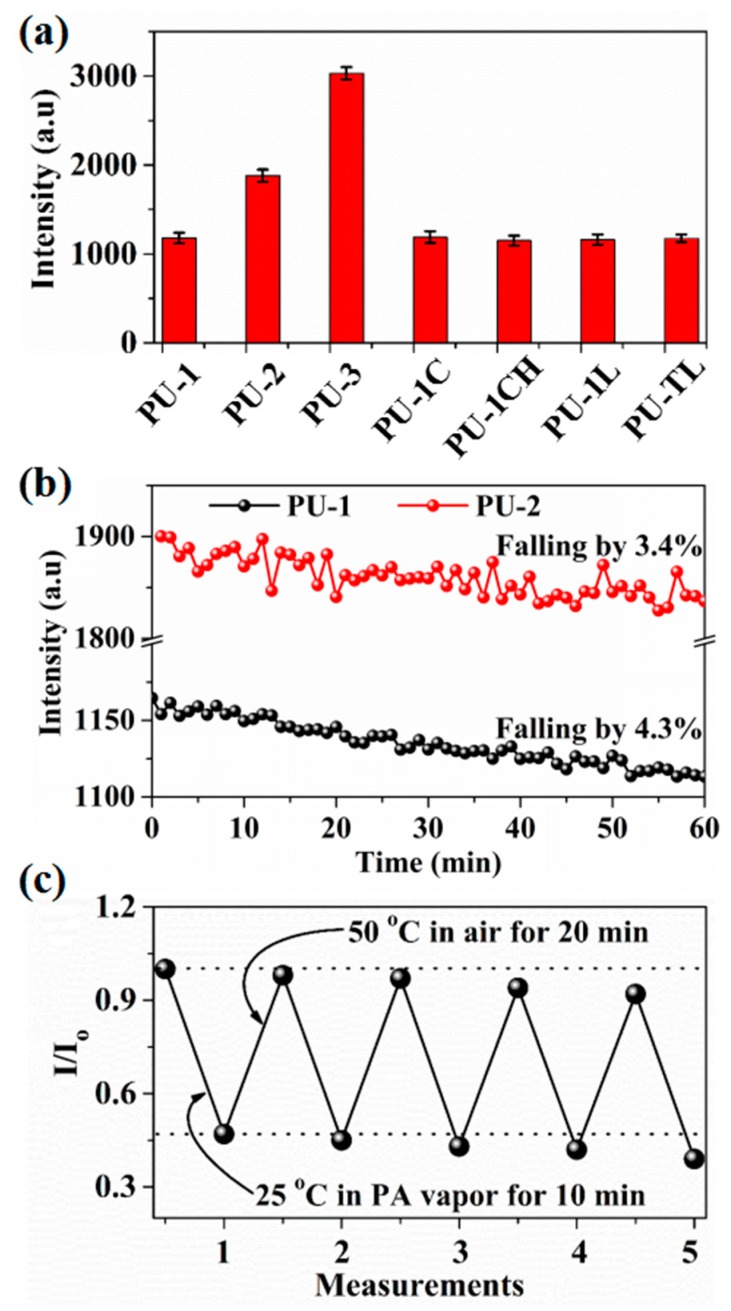
(**a**) Fluorescent homogeneity of 10 test points of fluorescent nanofibrous membranes (error bars represent the standard deviation of 10 test points of fluorescent membranes), (**b**) time stability of PU-1 and PU-2 membranes (relative humidity is 40%), (**c**) the normalized fluorescence recovery cycles of PU-1C membrane.

Homogeneity, stability and recoverability are known to be important for fluorescence sensors, therefore, these properties of fluorescent nanofibrous membranes are also evaluated. As shown in Figure 7a, the fluorescent homogeneity of 10 test points of fluorescent nanofibrous membranes is also investigated and both the modified nanofibrous membranes (PU-1C, PU-1CH, PU-1L and PU-1TL) and the pre-modified nanofibrous membranes (PU-1, PU-2, PU-3) show good fluorescent uniformity. The time stability of PU-1 and PU-2 membranes was tested at room temperature with the 40% relative humidity and the peak values at about 603 nm were collected every minute for 1 h (Figure 7b). Figure 7b shows that the fluorescent intensity of PU-1 and PU-2 membranes decreased by 4.3% and 3.4% after 1 h, respectively, showing the good time stability. The good time stability should be attributed to the UV irradiation treatment of the fluorescent nanofibrous membranes for 12 h to make the fluorescence intensity stable before quenching tests. Figure 7c illustrates the normalized fluorescence reversible investigation of PU-1C membrane with five cycles of PA detection between the saturated PA vapor at room temperature and the air at 50 °C. The PU-1L membrane shows a reversible fluorescent performance after five cycles of being immersed in the saturated PA vapor at room temperature for 10 min, heated in an oven at 50 °C for 20 min to detach the PA molecules. Evidently, this good recovery cycles demonstrate that the fluorescent nanofibrous membranes are reusable after a simple desorption process by heating. 

## 4. Conclusions

We have created and demonstrated the multichannel fluorescent sensor array based on nanofibrous membranes loaded with ZnS QDs for the discriminative detection of explosives. The array was constructed from nanofibrous membranes loaded with ZnS QDs featuring several surface ligands, playing a synergistic effect of the high surface-to-volume ratio of QDs, the good permeability of nanofiber membranes and the differential quenching introduced by surface ligands. The almost invariant transformation of fingerprints of four nitroaromatic explosives along with time was discovered. Four saturated vapors of nitroaromatic explosives could be reliably detected and discriminated by the array at room temperature. This work is the first step toward devising a monitoring system for explosives in the field of public security and defense. It could, for example, be coupled with the technology of image recognition and large data analysis for a rapid diagnostic test of explosives. This work further highlights the power of differential, multichannel arrays for the rapid and recognizable detection of a wide range of chemicals.

## Supplementary Materials

The following are available online at http://www.mdpi.com/1424-8220/17/11/2676/s1, Figure S1:Structural formula of lysine, cysteine, trifluoroacetyl lysine and cysteine hydrochloride, Figure S2: The infrared spectrum of ZnS QD, Figure S3: SEM images with different magnification of (a, b) pure PU nanofibrous membrane, the inset in (b) shows image of water drops and the corresponding contact angles, Figure S4: Schematic diagram of ZnS QDs loading on nanofiber surfaces, Figure S5: Reproducibility of the quenching sensitivity of the nanofibrous membranes, Figure S6: Time-dependent fluorescence curves of the sensing array based on fluorescent membranes towards saturated TNT, DNT and PA vapors at room temperature, Figure S7: Time-dependent fluorescence curves towards the saturated NB vapor at room temperature (a) PU-1C, (b) PU-1CH, (c) PU-1L and (d) PU-1TL, Figure S8: Structural formula of TNT, PA, DNT and NB.
