# Supplementary material for: Multichannel Discriminative Detection of Explosive Vapors with an Array of Nanofibrous Membranes Loaded with Quantum Dots
Source: Sensors (Basel). 2017 Nov 20;17(11):2676. doi: 10.3390/s17112676 (PMC5713073; doi:10.3390/s17112676)
Supplement: Supplementary file 1 [file sensors-17-02676-s001.doc]

**Supplementary Materials**

**Multichannel** **Discriminative Detection of Explosive Vapors with an Array of Nanofibrous Membranes Loaded with Quantum Dots**

Zhaofeng Wu,^1,2^ Haiming Duan,^1^ Zhijun Li,^1^* Jixi Guo,^2^ Furu Zhong,^2^ Yali Cao,^2^* Dianzeng Jia^2^*

^1^ School of Physics Science and Technology, Xinjiang University, Urumqi, Xinjiang 830046, China; [lizhjun@xju.edu.cn](mailto:lizhjun@xju.edu.cn)

^2^ Key Laboratory of Energy Materials Chemistry, Ministry of Education, Key Laboratory of Advanced Functional Materials, Xinjiang University, Urumqi, Xinjiang 830046, China; [caoyali@xju.edu.cn,](mailto:caoyali@xju.edu.cn,) [jdz@xju.edu.cn](mailto:jdz@xju.edu.cn)

***** Correspondence: [lizhjun@xju.edu.cn](mailto:lizhjun@xju.edu.cn),[caoyali@xju.edu.cn,](mailto:caoyali@xju.edu.cn,) [jdz@xju.edu.cn](mailto:jdz@xju.edu.cn); Tel.: +86-991-858-2401


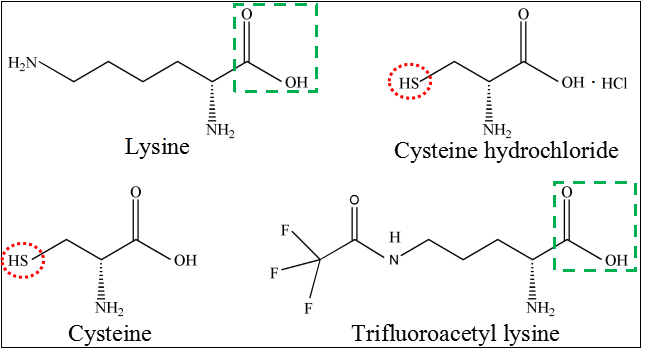


**Figure S1.** Structural formula of lysine, cysteine, trifluoroacetyl lysine and cysteine hydrochloride.

After modification, mercapto groups (as shown in red dotted coils in Figure S1) of cysteine and cysteine hydrochloride tightly attached onto the surface of the QDs due to the excess of metal ions with respect to sulfide ions at the surface of the QDs. Similarly, lysine and trifluoroacetyl lysine also tightly attached onto the surface of the QDs due to the interaction between carboxyl groups (as shown in the green dotted box in Figure S1) of lysine and trifluoroacetyl lysine and hydroxyl groups at the surface of the QDs.


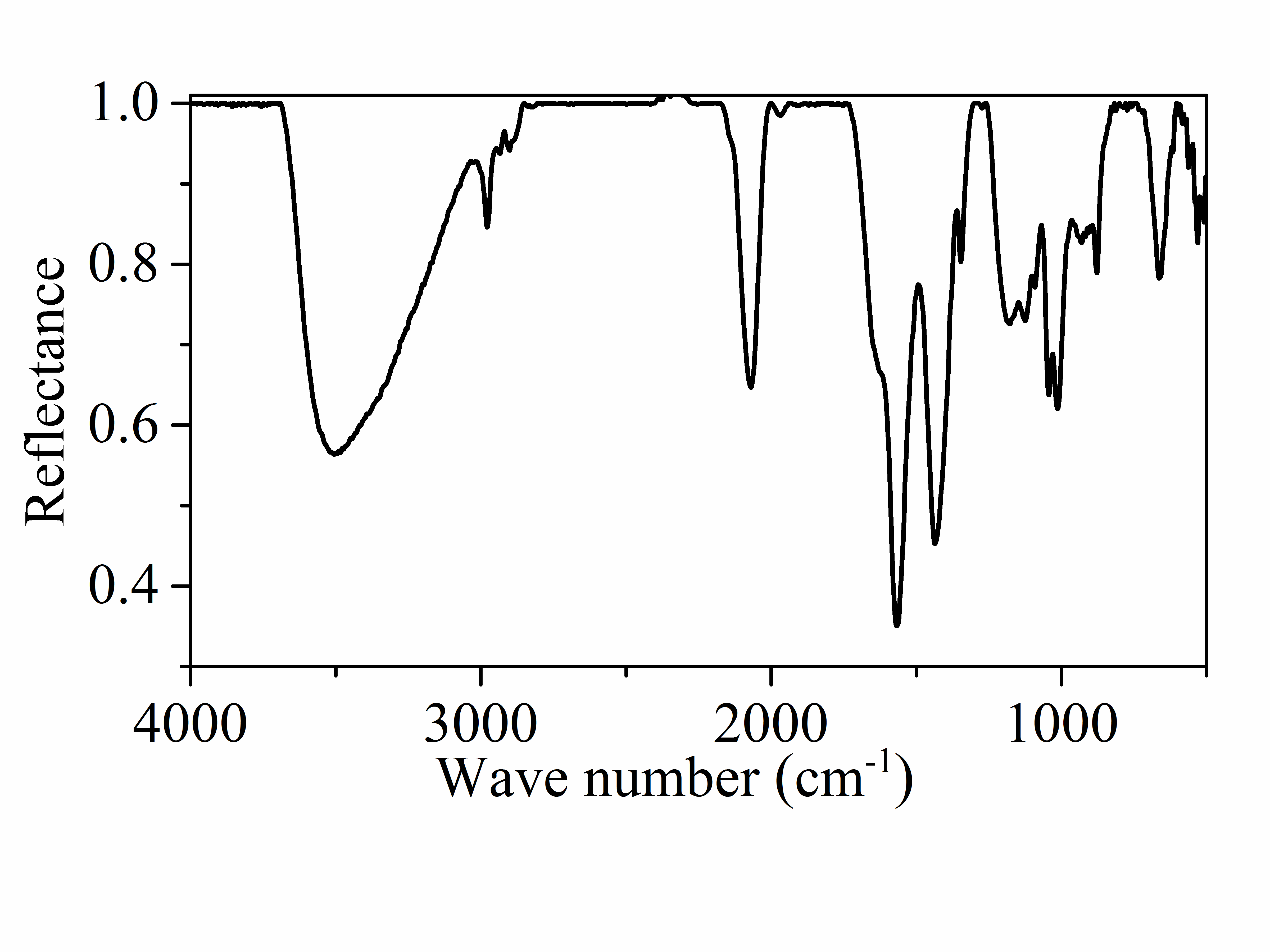


**Figure S2.** The infrared spectrum of ZnS QDs

As shown in Figure S2, the infrared bands located at about 3500 cm^-1^ are due to the -OH stretching on the surface ZnS QDs. The strong bands located at about 3500 cm^-1^ indicates the presence of a large number of hydroxyl on the surface of ZnS QDs.


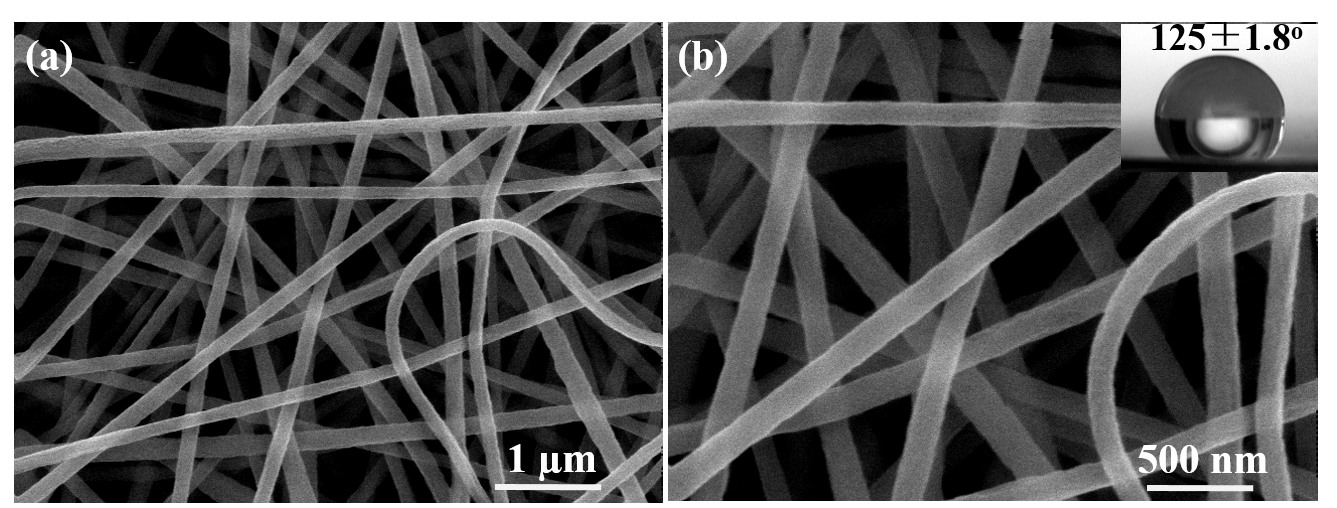


**Figure S3.** SEM images with different magnification of (a) (b) pure PU nanofibrous membrane, the inset in (b) shows image of water drops and the corresponding contact angles.


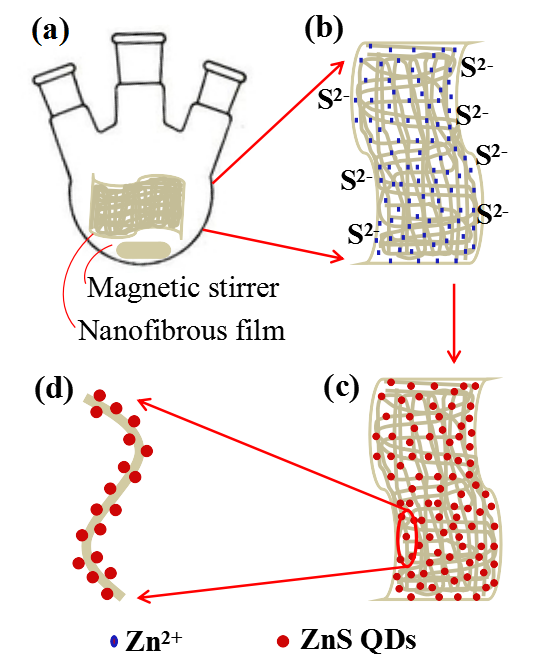


**Figure S4.** Schematic diagram of ZnS QDs loading on nanofiber surfaces.


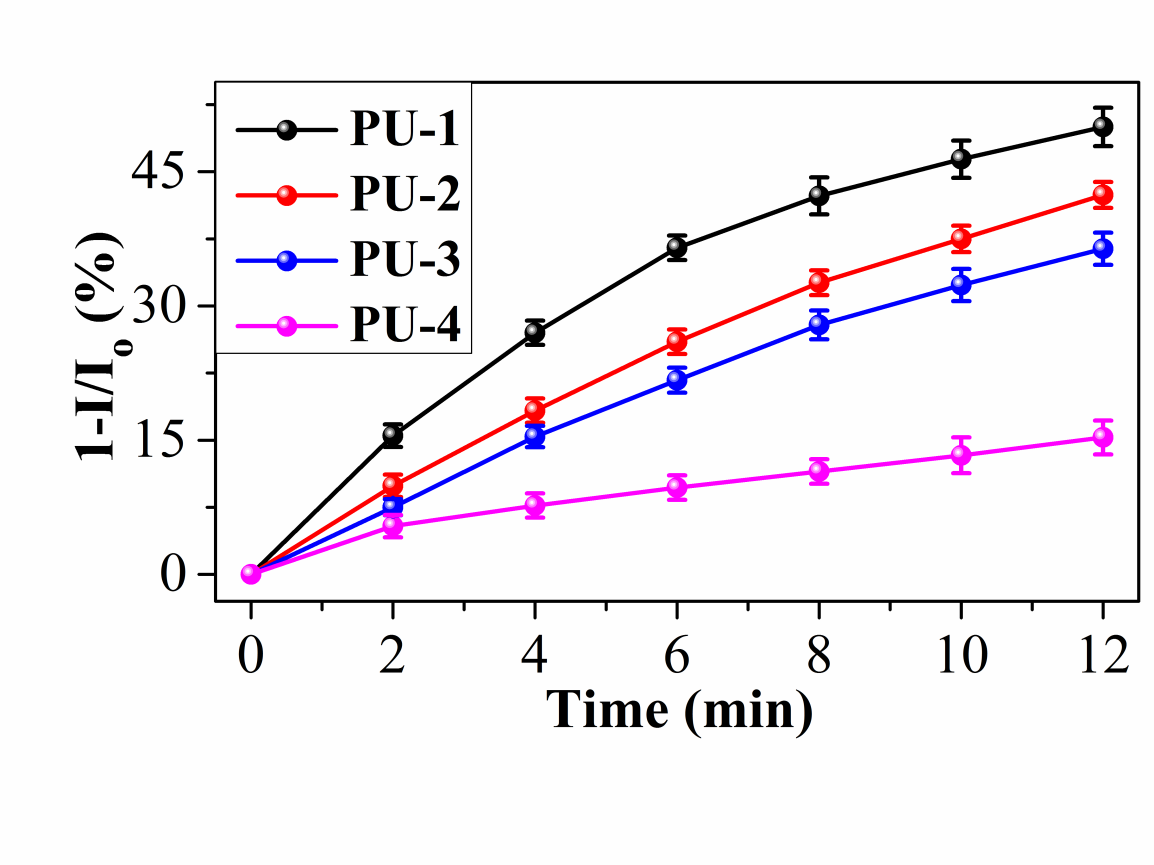


**Figure S5.** Reproducibility of the quenching sensitivity of the nanofibrous membranes. Error bars represent the standard deviation of three batches of nanofibrous membranes.


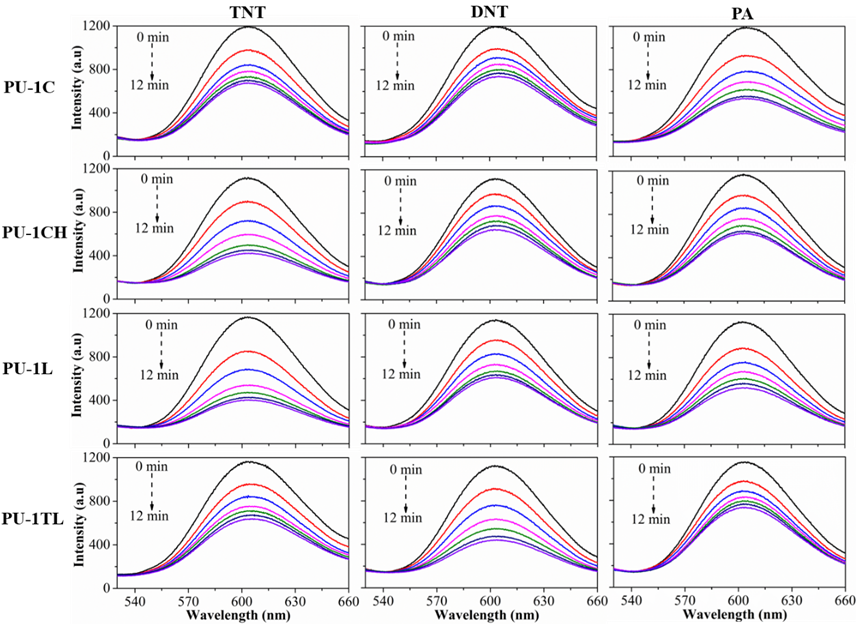


**Figure S6.** Time-dependent fluorescence curves of the sensing array based on fluorescent membranes towards saturated TNT, DNT and PA vapors at room temperature.


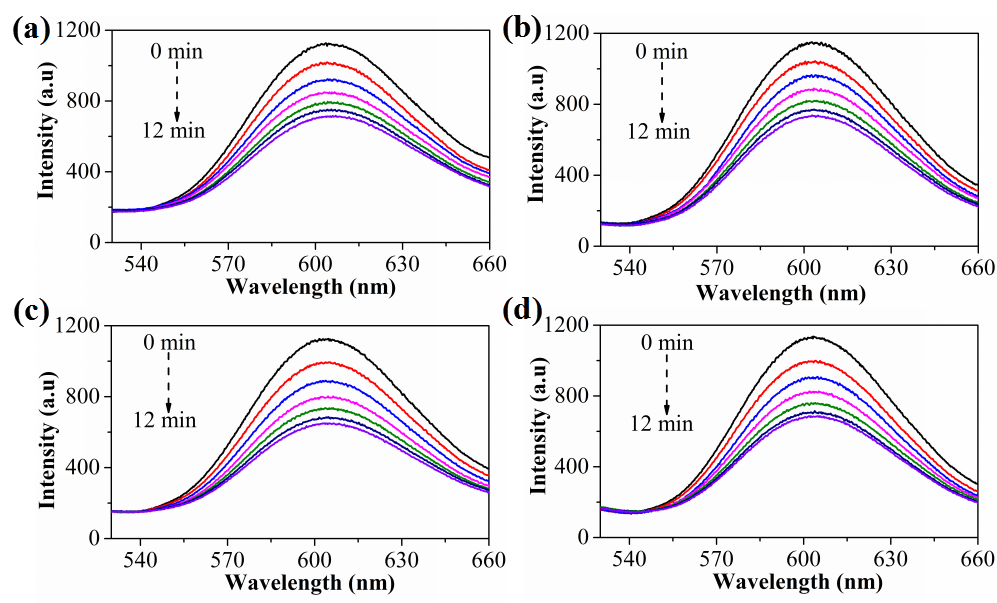


**Figure S7.** Time-dependent fluorescence curves towards the saturated NB vapor at room temperature (a) PU-1C, (b) PU-1CH, (c) PU-1L and (d) PU-1TL.


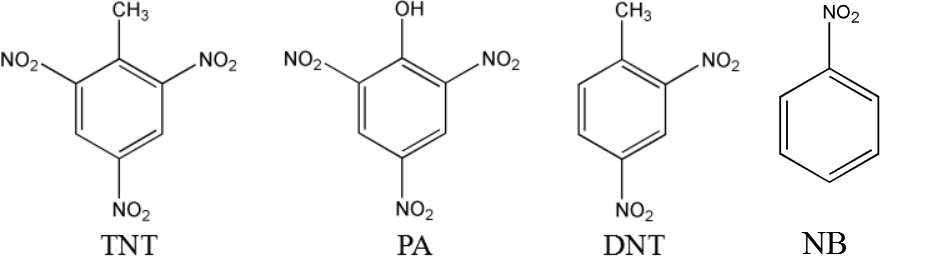


**Figure S8.** Structural formula of TNT, PA, DNT and NB.
